# High Expression of the SH3TC2-DT/SH3TC2 Gene Pair Associated With FLT3 Mutation and Poor Survival in Acute Myeloid Leukemia: An Integrated TCGA Analysis

**DOI:** 10.3389/fonc.2020.00829

**Published:** 2020-06-19

**Authors:** Pengfei Yu, Haifeng Lan, Xianmin Song, Zengkai Pan

**Affiliations:** ^1^Department of Hematology, Shanghai East Hospital, Tongji University School of Medicine, Shanghai, China; ^2^Hannover Medical School, Institute of Virology, Hanover, Germany; ^3^Department of Hematology, Shanghai General Hospital Affiliated to Shanghai Jiao Tong University, Shanghai, China; ^4^Department of Hematology, Hemostasis, Oncology, and Stem Cell Transplantation, Hannover Medical School, Hanover, Germany

**Keywords:** acute myeloid leukemia, divergent transcription, the cancer genome atlas, prognostic signature, FLT3 mutation

## Abstract

Fms-like tyrosine kinase 3 (FLT3) mutation is one of the most common mutations in acute myeloid leukemia (AML). However, the effect of FLT3 mutation on survival is currently still controversial and the leukemogenic mechanisms are still under further investigation. The aim of our study is to identify differentially expressed genes (DEGs) in FLT3-mutant AML and to find crucial DEGs whose expression level is related to prognosis for further analysis. By mining the TCGA-LAML dataset, 619 differentially expressed lncRNAs (DElncRNAs) and 1,428 differentially expressed mRNAs (DEmRNAs) were identified between FLT3-mutant and FLT3-wildtype samples. Through weighted gene correlation network analysis (WGCNA) and the following Cox proportional hazards regression analysis, we constructed the prognostic risk models to identify the hub DElncRNAs and DEmRNAs associated with AML prognosis. The presence of both SH3TC2 divergent transcript (SH3TC2-DT) and SH3TC2 in respective prognostic risk models promotes us to further study the significance of this gene pair in AML. SH3TC2-DT and SH3TC2 were identified to be coordinately high expressed in FLT3-mutant AML samples. High expression of this gene pair was associated with poor survival. Using logistic regression analysis, we found that high SH3TC2-DT/SH3TC2 expression was associated with FLT3 mutation, high WBC count, and intermediate cytogenetic and molecular–genetic risk. AML with SH3TC2-DT/SH3TC2 high expression showed enrichment of transcripts associated with stemness, quiescence, and leukemogenesis. Our study suggests that the SH3TC2-DT/SH3TC2 gene pair may be a possible biomarker to further optimize AML prognosis and may function in stemness or quiescence of FLT3-mutant leukemic stem cells (LSCs).

## Introduction

Acute myeloid leukemia (AML) is a group of clonal diseases that are characterized by heterogeneously genetic or epigenetic mutations. Fms-like tyrosine kinase 3 (FLT3) gene mutation is frequently found in adult AML. Two types of FLT3 activating mutations have been identified: internal tandem duplication (ITD) mutation and tyrosine kinase domain (TKD) point mutation. Twenty to thirty percent of adult AML patients have ITD mutation and 5–10% of them have TKD mutation (1, 2). FLT3 activating mutations were considered to result in constitutive activation of FLT3 and downstream signaling pathways to promote growth and survival of leukemic cells. However, to emphasize, the mechanism of mutant FLT3 activation in leukemogenesis has not been definitely confirmed. In clinical setting, FLT3 mutation has been closely related to leukocytosis and high blast percentage in marrow (3, 4). However, the prognostic significance of FLT3 mutation is still controversial. The prognostic value of FLT3-ITD mutation was reported to be associated with ITD allelic ratio and the presence of NPM1 mutation (1). Although ELN 2017 recommendation indicates that adult patients with concurrent mutant NPM1 and FLT3-ITD of low allelic ratio (<0.5) have favorable outcome (1), another study suggests that these patients have inferior survival compared to other favorable-risk AML patients, and concurrent DNMT3A mutation may serve as a risk modifier (5). The effect of FLT3-TKD mutation on survival is currently still unclear. Recent studies showed that FLT3-TKD mutation was associated with favorable outcome in the presence of NPM1 mutation (6), but associated with poor outcome in the presence of MLL-PTD (7). To further elucidate the potential genes in association with FLT3 mutation and prognosis of AML is meaningful.

Over these years, the genomic and transcriptomic features of AML have been comprehensively characterized by next-generation sequencing. Several publicly available databases from large patient cohorts have been constructed, which provide the opportunity to identify biomarkers in correlation with disease development, evolution, and treatment response. Identification of these potentially critical genes could provide hints for validation and clinical application (3, 4).

In this study, using a series of bioinformatic tools, we identified for the first time that the SH3TC2-DT/SH3TC2 gene pair was highly expressed in FLT3-mutant AML and presented as crucial genes associated with prognosis. The SH3TC2-DT/SH3TC2 gene pair may play a role in stemness or quiescence of FLT3-mutant LSCs. Our study provides the basis to further analyze the function of the SH3TC2-DT/SH3TC2 gene pair in the development and prognosis of FLT3-mutant AML.

## Materials and Methods

### Data Collection and Preprocessing

**Figure 1:**
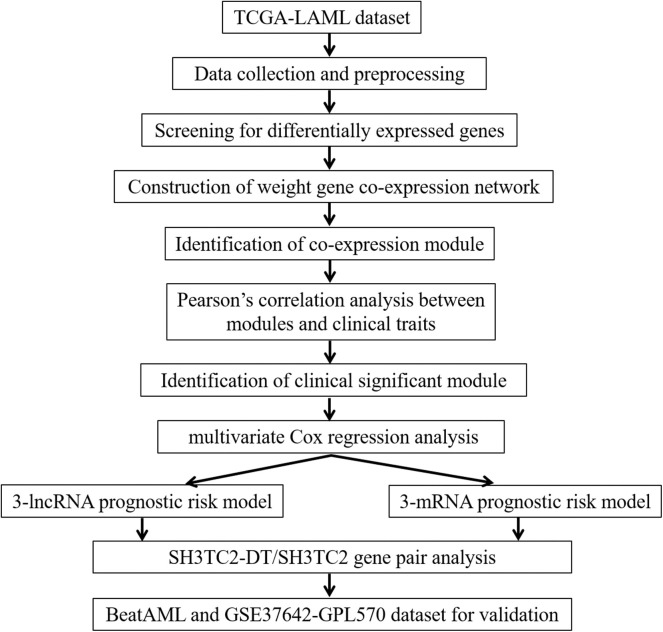
Workflow chart of this study.

A workflow chart of this study is shown in Figure 1. The data of 151 human AML samples used in this study were downloaded from The Cancer Genome Atlas (TCGA) database (https://portal.gdc.cancer.gov/), including the RNA sequencing data derived from the IlluminaHiSeq_RNASeq platform and clinical follow-up data, such as age, blast percentage, and survival time (data status as of Sept. 26, 2018) (3).

### Identification of Differentially Expressed lncRNAs and mRNAs

The TCGA-LAML dataset consists of 43 FLT3-mutant AML and 108 FLT3-wildtype AML samples. R package “edgeR” was used to screen for differentially expressed genes (DEGs) between FLT3-mutant and FLT3-wildtype samples. The *q*-values use false discovery rate (FDR) to adjust the statistical significance of multiple hypothesis testing. Fold change (FC) ≥2 and adjusted *P* < 0.05 were considered to be statistically significant (8–10). The ensemble ID was switched to gene symbol according to the human genome assembly GRCh38.93. Afterwards, for DElncRNAs and DEmRNAs, volcano maps were depicted by “gplots” package in the R platform.

### Functional Enrichment Analysis

R package “clusterProfiler” was used for Kyoto Encyclopedia of Genes and Genomes (KEGG) analysis (11, 12). Enriched pathways with the significant level at *P* < 0.05 were selected. Gene-set enrichment analysis (GSEA) was used to identify the significant gene sets enriched in SH3TC2-DT or SH3TC2 high-expression phenotype (13, 14).

### Weighted Gene Co-expression Network Analysis

R package “WGCNA” was used to construct co-expression modules for DEGs (15). Briefly, by “hclust” method to evaluate the expression matrix, the sample of TCGA-AB-2935 was removed from further analysis, because it was considered to be outliers as the cluster height was over 3 × 10^5^ (as shown in Figure S1). The other samples of TCGA-LAML were clustered by methods of average linkage and Pearson's correlation. The weighted adjacency matrix between genes *i* and *j* was defined as a*ij* = |C*ij*|^β^ (a*ij*: adjacency between gene *i* and gene *j*, C*ij*: the Pearson's correlation, β: soft-power threshold = 4). Afterwards, the clinical traits were accessed.

Three samples (TCGA-AB-2946, TCGA-AB-2895, and TCGA-AB-2810) were excluded from further analyses due to lack of clinical data. The scale independence and mean connectivity were calculated. Then, a topological overlap matrix (TOM) was transformed from the adjacency matrix (16). Finally, according to the TOM-based dissimilarity measure with a minimum size threshold of 30, average linkage hierarchical clustering dendrogram was constructed to classify genes with similar expression profiles into the same modules using the DynamicTreeCut algorithm. To identify the clinical significance of each module, gene significance (GS) was calculated to quantify associations of individual gene with clinical trait. Module significance (MS) was defined as the association between the module eigengenes (MEs) and the gene expression profiles. The different MEs were then correlated to the clinical traits (17, 18).

### Cox Proportional Hazards Regression Analysis

The prognostic significance of each yellow module gene was evaluated by univariate Cox proportional hazards regression. Kaplan–Meier curve was depicted for each gene using R package “survival.” Afterwards, multivariate Cox regression analysis was used to construct a 3-lncRNA prognostic risk model from prognosis-related lncRNAs. As to mRNA, least absolute shrinkage and selection operator (LASSO) regression analysis was first performed to select mRNAs in order to enhance the prediction accuracy of the prognostic risk model. Then, multivariate Cox regression analysis was used to construct a 3-mRNA prognostic risk model from the selected mRNAs. The AML samples were separated into high-risk and low-risk groups according to the median of the risk score. The risk score and survival status of each sample were depicted. Heatmap was generated by R package “pheatmap” to show the expression of risk genes in each sample. Kaplan–Meier analysis was conducted to identify the prognostic value of the risk model. The receiver operating characteristic (ROC) curve was used to evaluate the accuracy of the risk model or gene by R package “survivalROC.” Nomogram was drawn to predict overall survival (OS) by the results of the multivariate Cox regression analysis.

### SH3TC2-DT/SH3TC2 Gene Pair Analysis

For single gene analysis, the Student's *t*-test was used for expression comparison. Survival curve, ROC curve, and multivariate Cox regression analysis were performed as previously described. Logistic regression was used to analyze the association between the SH3TC2-DT/SH3TC2 expression and clinical characteristics.

To predict the potential targets of SH3TC2, we firstly analyzed the DEGs between SH3TC2 high-expression group (*n* = 76) and SH3TC2 low expression group (*n* = 75) by R package “edgeR.” Then, DEGs list was annotated by the module “UCSC_TFBS” under the “Protein_Interactions” function of DAVID (19, 20). Significantly enriched transcription factors (TFs) in DEGs were identified (adjusted *P* < 0.05). The TFs network was visualized by Cytoscape (3.7.1).

The RNA sequencing data and clinical follow-up data of BeatAML dataset were downloaded from Vizome (http://www.vizome.org/) (21) and TCGA (https://portal.gdc.cancer.gov/) to validate the differential expression of SH3TC2-DT/SH3TC2 between FLT3-ITD and FLT3-wildtype AML. DEGs were calculated as previously described. The GSE37642-GPL570 AML dataset (22) was used to validate the association between SH3TC2 expression level and OS. AML survival and microarray data were downloaded from the GEO platform (https://www.ncbi.nlm.nih.gov/geo/). The 136 AML samples were separated into two groups according to the median of SH3TC2 expression level. Kaplan–Meier curve was used to compare the OS between high (*n* = 68) and low (*n* = 68) SH3TC2 expression AML samples.

All statistical tests and graphing were performed by R and GraphPad Prism 7.0. *P* < 0.05 was considered of statistical significance. In the figures, statistical significance was shown as follows: ^*^P < 0.05, ^**^P < 0.01, ^***^P < 0.001, and ^****^P < 0.0001.

## Results

### DEmRNAs and DElncRNAs Between FLT3-Mutant and FLT3-Wildtype AML

**Figure 2:**
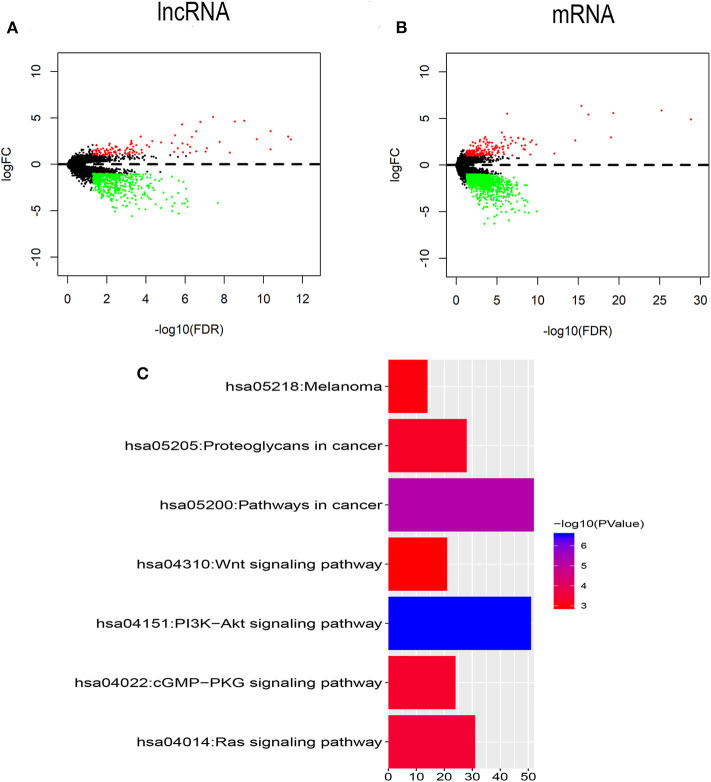
Identification of DElncRNAs and DEmRNAs between FLT3-mutant and FLT3-wildtype AML. **(A,B)** Volcano plots of DElncRNAs **(A)** and DEmRNAs **(B)**. The cutoff values are FC> 2 and FDR< 0.05. Red and green dots indicate upregulated and downregulated RNAs, respectively. Black dots denote no difference in RNA expression. FC, fold change; FDR, false discovery rate; lncRNA, long non-coding RNA; mRNA, messenger RNA; DElncRNAs, differentially expressed lncRNAs; DEmRNAs, differentially expressed mRNAs. **(C)** KEGG pathways in relation with tumorigenesis enriched for DEmRNAs are shown. *X* axis showing the number of DEmRNAs enriched in each pathway. KEGG, Kyoto Encyclopedia of Genes and Genomes.

To identify the transcriptomic features related to FLT3 mutation, R package “edgeR” was used to perform differential expression analysis in TCGA-LAML dataset. Compared with FLT3-wildtype AML, 619 lncRNAs (113 upregulated and 506 downregulated) and 1,428 mRNAs (194 upregulated and 1,234 downregulated) were significantly differentially expressed in FLT3-mutated AML (FC> 2, FDR< 0.05) (Figures 2A,B). KEGG analysis revealed that DEmRNAs were enriched in pathways closely related to tumorigenesis, such as Wnt signaling pathway, PI3K-Akt signaling pathway, and Ras signaling pathway (Figure 2C), suggesting the possible function of FLT3 mutation in AML pathogenesis.

### Construction of Weighted Co-expression Network and Identification of Survival-Associated Module

**Figure 3:**
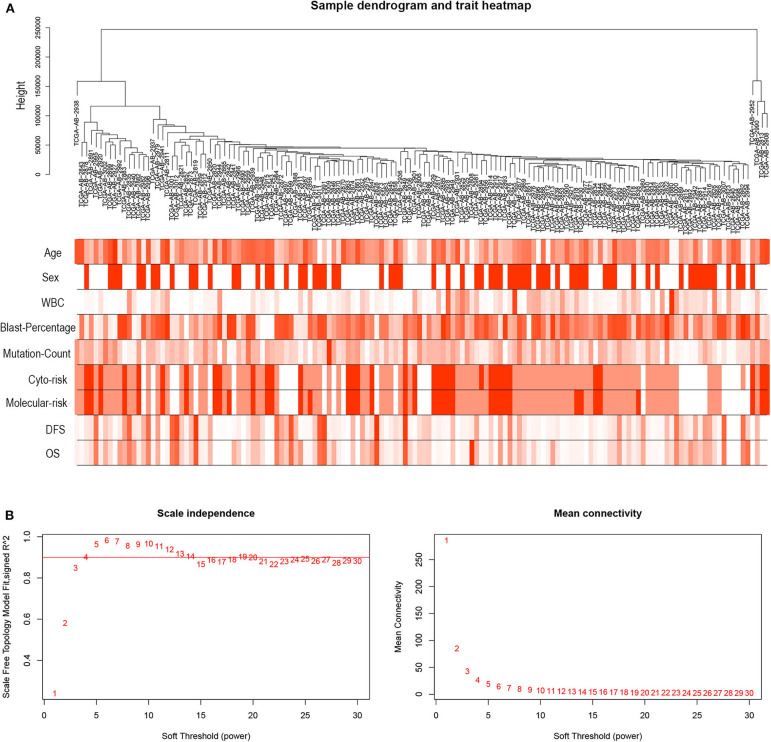
Hierarchical clustering tree and soft-thresholding values estimation. **(A)** Hierarchical clustering tree of the TCGA-LAML samples. Dendrogram tips are denoted with TCGA-LAML sample names. The nine bands below dendrogram indicate clinical traits of each sample. WBC, white blood cell; Cyto-risk, cytogenetic risk; Molecular-risk, molecular genetic risk; DFS, disease-free survival; OS, overall survival. **(B)** Scale independence and mean connectivity across different soft-thresholding values (β).

**Figure 4:**
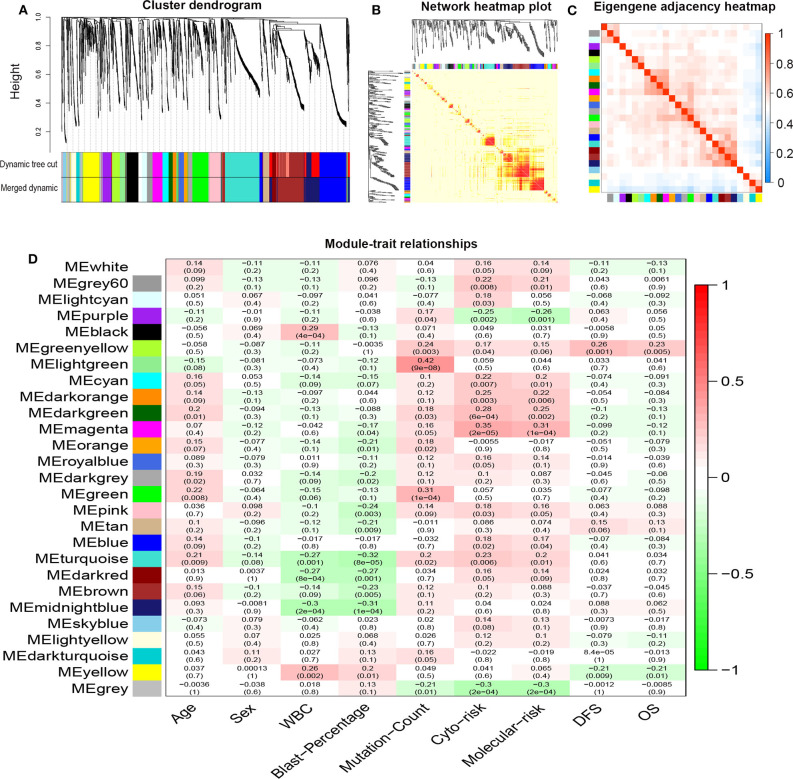
Network construction of co-expressed genes and module–trait relationships. **(A)** Cluster dendrogram and the co-expression network modules produced by average linkage hierarchical clustering of DEGs based on topological overlaps. Each branch within the dendrogram indicates a single gene. Height depicts the Euclidean distance. Each color indicates a single module that contains weighted co-expressed genes. **(B)** Heatmap view of topological overlap. Four hundred randomly selected genes grouped into modules displayed color codes, which are shown beneath the cluster dendrogram. Dark yellow and red represent a high degree of topological overlap. **(C)** Eigengene adjacency heatmap. The heatmap represents the relationship among distinctive co-expression modules. **(D)** Module–trait relationships. Each row represents a color module and each column indicates a clinical trait. Each cell contains *R*^2^-values of Pearson correlations between the modules and clinical features and the corresponding *P*-value in parentheses. Gradient color of each cell indicates the *R*^2^-values of Pearson correlations (red = 1, green = −1). DEGs, differentially expressed genes; ME, module eigengene.

R package “WGCNA” was used to construct co-expression modules of DEGs and to further identify prognosis-related modules. First, the samples of TCGA-LAML were clustered by methods of average linkage and Pearson's correlation (Figure 3A). To ensure a scale-free network, β = 4 (scale free *R*^2^ = 0.9) was set as the soft-thresholding parameter (Figure 3B). After merging modules with high similarity, a total of 27 modules with the size ranging from 31 to 327 genes were generated by the average linkage hierarchical clustering (Figure 4A). The non-co-expressed genes were grouped into “gray” module and removed from further analysis. The heatmap of 400 randomly selected DEGs showed high degree of topological overlap of co-expressed genes in each module (Figure 4B). The eigengene adjacency heatmap showed relationships between the 27 co-expression modules (Figure 4C). At last, the correlation between these modules and clinical traits was determined (Figure 4D). The yellow module was correlated with high white blood cell (WBC) count [Pearson correlation coefficient (PCC) = 0.26, *P* = 0.002] and blast percentage in bone marrow (PCC = 0.2, *P* = 0.01), but not correlated with age, sex, mutation count, cytogenetic risk, or molecular–genetic risk. Of note, this module had the highest association with worse disease-free survival (DFS) (PCC = −0.21, *P* = 0.009) and OS (PCC = −0.21, *P* = 0.01), and was selected for further analysis.

### Prognostic Significance of Each Gene in the Yellow Module

**Figure 5:**
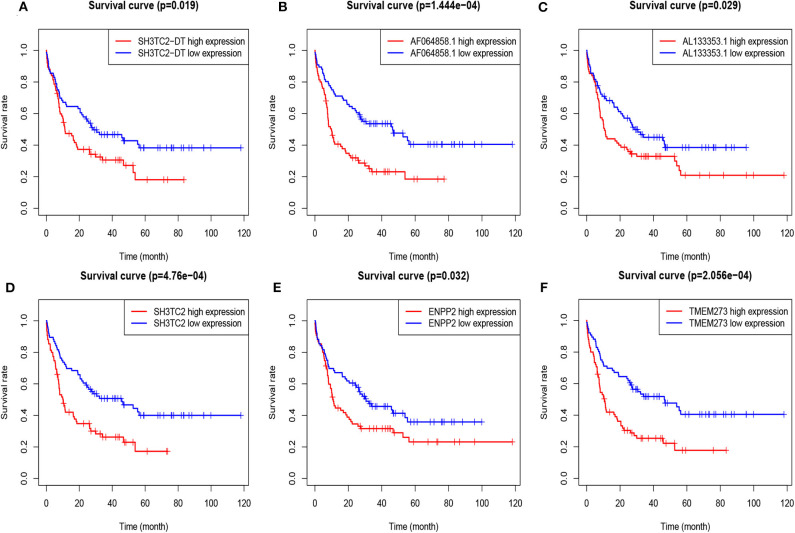
Overall survival analysis of TCGA-LAML cohort based on expression of yellow module genes by Kaplan–Meier plotter. The patients were grouped into high-expression and low-expression group based on median expression of each gene. Survival plots of selected lncRNAs **(A–C)** and mRNAs **(D–F)** are shown.

A total of 43 genes in the yellow module were identified to be significantly related to OS by univariate Cox proportional hazards regression. Among them, high expression of 12 lncRNAs (SH3TC2-DT, LINC00982, LINC00899, GRM7-AS1, MIR155HG, AC005392.2, LINC01132, AL133353.1, AF064858.1, LINC01979, AC103702.1, and DLGAP1-AS3) and 31 mRNAs (TMEM273, PRDM16, SH3TC2, LCT, ENPP2, CCDC113, ATRNL1, TRIM16, LDLRAD2, MPZL2, HOXB6, SCHIP1, ARHGEF5, CAMK2A, NLRP2, CCL1, H2AFY2, GLI2, APOL4, HOXA7, PBX3, PDGFD, HOXB2, HOXB5, LCN8, TRIM15, GOLGA8B, CACNG4, FAM47E-STBD1, REN, and FAM47E) were associated with poor OS (Figure 5, Tables S1, S2). These lncRNAs and mRNAs were then subjected to further construct lncRNA or mRNA prognostic risk model.

### Establishment of the lncRNA Prognostic Risk Model

**Figure 6:**
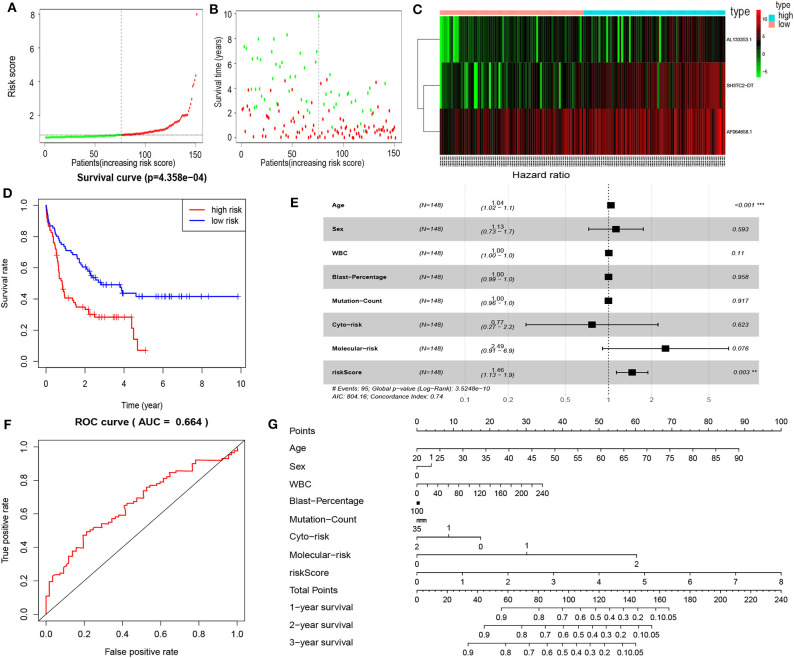
Cox proportional hazards regression analysis of lncRNAs. **(A)** The lncRNA risk score distribution of TCGA-LAML samples. **(B)** Survival status and time of all AML patients across lncRNA risk score. The AML patients were separated into two groups according to the median of risk score. Red dots indicate high-risk samples (*N* = 74). Green dots indicate low-risk samples (*N* = 74). **(C)** Heatmap and hierarchical clustering of lncRNAs from the prognostic risk model were generated. Values are normalized with log_10_. The right longitudinal axis: the names of lncRNAs; the left longitudinal axis: the clustering information of lncRNAs. Red and green denote upregulated and downregulated lncRNAs, respectively. high, high-risk samples; low, low-risk samples. **(D)** Kaplan–Meier curve to compare OS of high-risk with low-risk samples (*P* = 4.358e−04). **(E)** Forest plot of multivariable Cox regression analysis. The squares on the transverse lines show the hazard ratio (HR), and the transverse lines represent 95% confidence interval (CI). **(F)** ROC curves showing predictive value of the prognostic risk model. **(G)** Nomogram of OS prediction in AML. **P < 0.01; ***P < 0.001.

By multivariate Cox proportional hazards regression analysis, we built a 3-lncRNA prognostic risk model to predict OS in AML cases as follows: risk score = (0.006899 × expression level of SH3TC2-DT) + (0.00026 × expression level of AF064858.1) + (0.016446 × expression level of AL133353.1) (Table 1). Of note, SH3TC2-DT is the most significant prognosis-associated lncRNA in this model (*P* = 0.000234) (Table 1). A total of 148 patients were categorized into high-risk (*N* = 74) and low-risk (*N* = 74) group according to the median of risk score (Figures 6A–C). The OS rate of high-risk patients was significantly lower compared with low-risk patients (Figure 6D). Multivariate Cox regression analysis revealed that age and the lncRNA risk score were independent prognostic factors affecting OS. The lncRNA risk score had a greater influence on survival than WBC count, cytogenetic risk, and molecular risk (Figure 6E). The area under ROC curve was 0.664, showing a high predictive value of the risk model (Figure 6F). Nomogram was drawn to visualize the result of multivariate Cox regression analysis (Figure 6G). Furthermore, Kaplan–Meier curves also confirmed that these three lncRNAs were predictive indicators for OS (Figures 5A–C).

**Table 1:** lncRNA prognostic risk score model.

**lncRNA**	**Coefficient**	**HR in OS**	***P*-value**
SH3TC2-DT	0.006899	1.0069 (1.0032–1.0106)	0.0002
AF064858.1	0.00026	1.0003 (1.0000–1.0005)	0.0386
AL133353.1	0.016446	1.0166 (0.9991–1.0344)	0.0637

*HR, hazard ratio; OS, overall survival. Numbers in parentheses show the 95% CI (confidence interval) of HR*.

### Establishment of the mRNA Prognostic Risk Model

**Figure 7:**
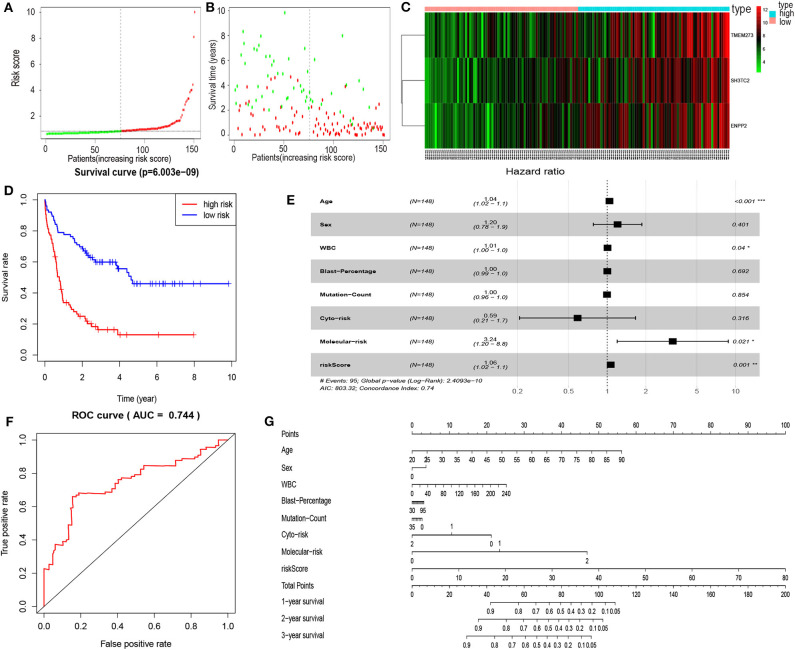
Cox proportional hazards regression analysis of mRNAs. **(A)** The mRNA risk score distribution of TCGA-LAML samples. **(B)** Survival status and time of all AML patients across mRNA risk score. The AML patients were separated into two groups according to the median of risk score. Red dots indicate high-risk samples (*N* = 74). Green dots indicate low-risk samples (*N* = 74). **(C)** Heatmap and hierarchical clustering of the mRNAs in prognostic risk model were generated. Values used are normalized with log_10_. The right longitudinal axis: the names of mRNAs; the left longitudinal axis: the clustering information of mRNAs. Red and green denote upregulated and downregulated mRNAs, respectively. high, high-risk samples; low, low-risk samples. **(D)** Kaplan–Meier curve to compare the OS of high-risk and low-risk samples (*P* = 6.003e−09). **(E)** Forest plot of multivariable Cox regression analysis. The squares on the transverse lines show the HR, and the transverse lines represent 95% CI. **(F)** ROC curves showing predictive value of the prognostic risk model. **(G)** Nomogram of OS prediction in AML. *P < 0.05; **P < 0.01; ***P < 0.001.

In order to enhance the prediction accuracy of the prognostic risk model, we firstly performed LASSO regression analysis and selected four mRNAs (SH3TC2, ENPP2, TMEM273, and PRDM16) for further analysis from the 31 mRNAs with prognostic value in yellow module (Figure S2). Afterwards, through multivariate Cox proportional hazards regression analysis, we identified a 3-mRNA prognostic risk model to predict OS in AML cases as follows: risk score= (0.000612 × expression level of SH3TC2) + (0.000507 × expression level of ENPP2) + (0.000277 × expression level of TMEM273) (Table 2). A total of 148 patients were categorized into high-risk (*N* = 74) and low-risk (*N* = 74) group according to the median of risk score (Figures 7A–C). The OS rate of high-risk patients was significantly lower compared with low-risk patients (Figure 7D). Multivariate Cox regression analysis revealed that age, WBC count, molecular risk, and the mRNA risk score were independent prognostic factors affecting OS. The mRNA risk score had a greater influence on survival than WBC count, cytogenetic risk, and molecular risk (Figure 7E). The area under ROC curve was 0.744, showing a high predictive value of the risk model (Figure 7F). Finally, Nomogram was drawn to visualize the result of multivariate Cox regression analysis (Figure 7G). Furthermore, Kaplan–Meier curves also confirmed that these three mRNAs were predictive indicators for OS (Figures 5D–F).

**Table 2:** mRNA prognostic risk score model.

**mRNA**	**Coefficient**	**HR in OS**	***P*-value**
SH3TC2	0.000612	1.0006 (1.0002–1.0010)	0.0016
ENPP2	0.000507	1.0005 (1.0002–1.0009)	0.0042
TMEM273	0.000277	1.0003 (1.0001–1.0004)	0.0007

*HR, hazard ratio; OS, overall survival. Numbers in parentheses show the 95% CI (confidence interval) of HR*.

### The SH3TC2-DT/SH3TC2 Gene Pair Is an Independent Prognostic Factor for AML

**Figure 8:**
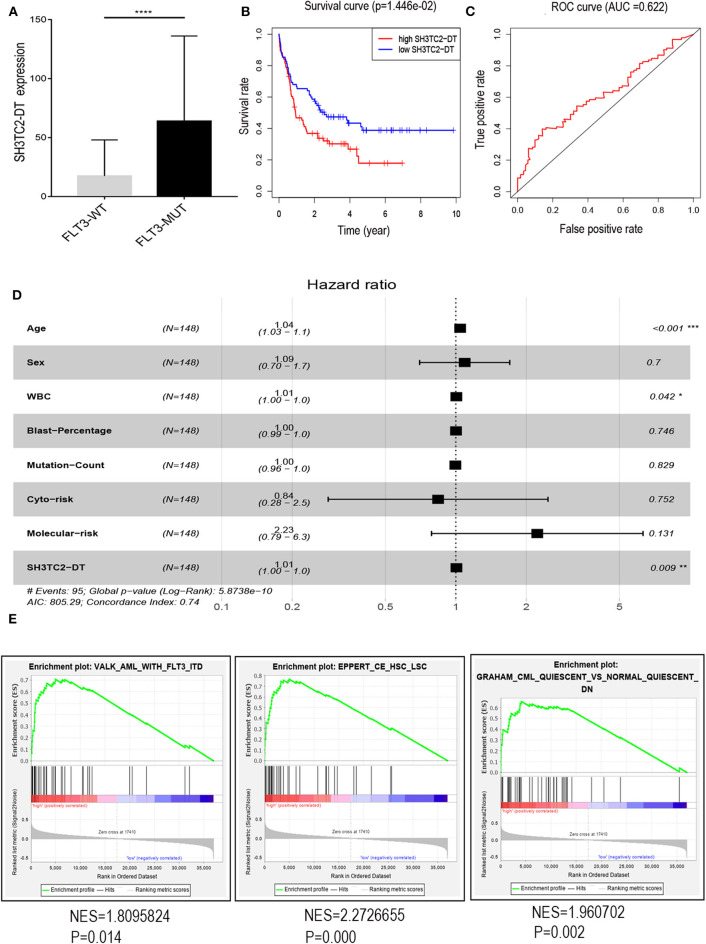
The association of SH3TC2-DT expression with overall survival and gene-set enrichment. **(A)** The normalized RNA-sequencing expression value of SH3TC2-DT in FLT3-WT (*N* = 108) and FLT3-MUT (*N* = 43) AML patients. **(B)** Kaplan–Meier curve to compare OS between high-SH3TC2-DT (*N* = 74) and low-SH3TC2-DT (*N* = 74) samples. The AML patients were separated into two groups according to the median of SH3TC2-DT expression level. **(C)** ROC curves showing predictive value of the SH3TC2-DT expression. **(D)** Forest plot of multivariable Cox regression analysis. **(E)** GSEA identified gene sets enriched in SH3TC2-DT high-expression phenotype. FLT3-WT, FLT3-wildtype; FLT3-MUT, FLT3-mutant. NES, normalized enrichment score. *P < 0.05; **P < 0.01; ***P < 0.001; ****P < 0.0001.

**Figure 9:**
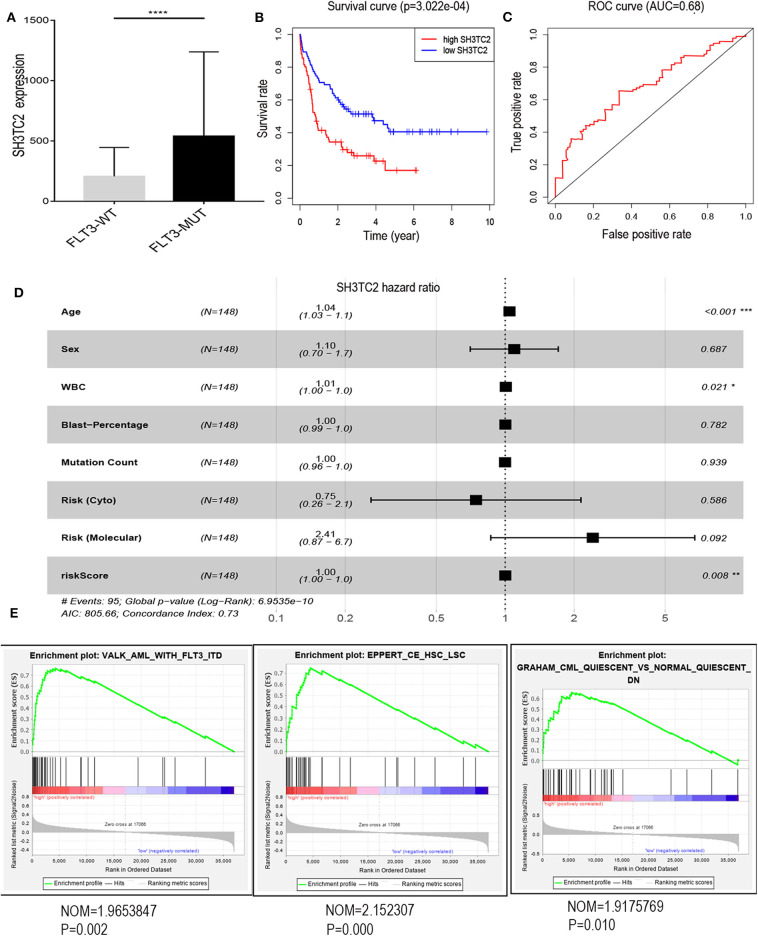
The association of SH3TC2 expression with overall survival and gene-set enrichment. **(A)** The normalized RNA-sequencing expression value of SH3TC2 in FLT3-WT (*N* = 108) and FLT3-MUT (*N* = 43) AML patients. **(B)** Kaplan–Meier curve to compare OS between high-SH3TC2 (*N* = 74) and low-SH3TC2 (*N* = 74) samples. The AML patients were separated into two groups according to the median of SH3TC2 expression level. **(C)** ROC curves showing predictive value of the SH3TC2 expression. **(D)** Forest plot of multivariable Cox regression analysis. **(E)** GSEA identified gene sets enriched in SH3TC2 high expression phenotype. *P < 0.05; **P < 0.01; ***P < 0.001; ****P < 0.0001.

**Figure 10:**
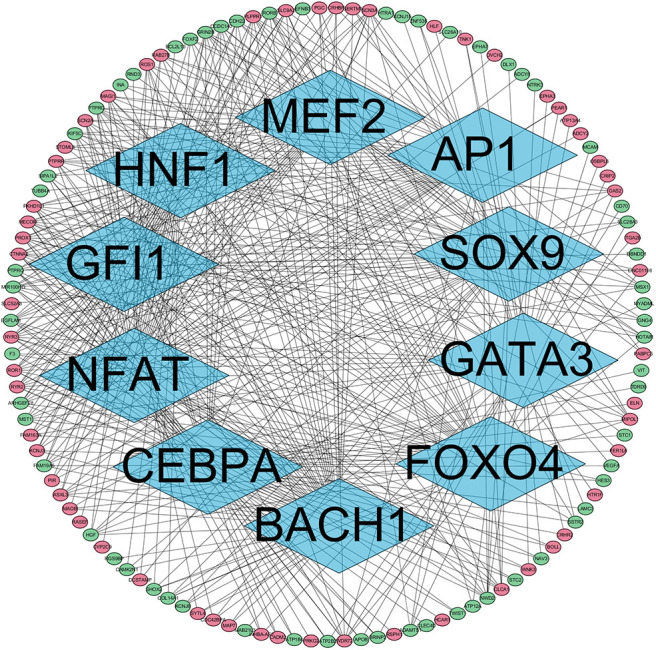
The TFs regulatory network enriched for DEGs between SH3TC2 high-expression AML and SH3TC2 low-expression AML. Diamonds, TFs; ellipses, genes regulated by TFs; red, high expressed genes; green, low expressed genes. TFs associated with stemness and leukemogenesis were selected to shown.

The presence of SH3TC2-DT and SH3TC2 in respective prognostic risk models emphasized the importance of this divergent lncRNA/mRNA gene pair in prognosis of FLT3-mutant AML (Tables 1, 2), but interestingly, the expression of the SH3TC2-DT/SH3TC2 gene pair showed no significant difference between FLT3-ITD and FLT3-TKD AML samples (Figure S3). As the general importance of divergent transcription is poorly understood, we further studied the clinical significance of SH3TC2-DT and SH3TC2 expression in TCGA-LAML cohort. The previous finding that divergent lncRNA/mRNA pairs exhibited coordinate changes in transcription suggests that divergent transcript might regulate gene transcription (23, 24). Our study revealed that SH3TC2-DT and SH3TC2 were also coordinately high expressed in FLT3-mutant AML samples (Figures 8A, 9A), suggesting that SH3TC2-DT might regulate SH3TC2 expression during AML pathogenesis. High expression of SH3TC2-DT or SH3TC2 was associated with poor OS (Figures 8B, 9B). The area under ROC curve was 0.622 for SH3TC2-DT and 0.68 for SH3TC2, both showing a high predictive value (Figures 8C, 9C). Multivariate Cox regression analyses revealed that both SH3TC2-DT and SH3TC2 expression were independent prognostic factors (Figures 8D, 9D). Furthermore, we used logistic regression analysis to relate SH3TC2-DT/SH3TC2 gene pair with clinical features and revealed that both high expression of SH3TC2-DT and SH3TC2 were associated with higher WBC count, intermediate cytogenetic and molecular–genetic risk, and FLT3 mutation. The SH3TC2 high expression was also associated with older age (Tables 3, 4). GSEA showed that gene sets of AML with FLT3-ITD, HSC (hematopoietic stem cell)/LSC and CML (chronic myelocytic leukemia) quiescence were enriched in both SH3TC2-DT and SH3TC2 high-expression phenotype (Figures 8E, 9E). We found that TFs associated with stemness (e.g., MEF2, AP1, SOX9, GATA3, and GFI1) or leukemogenesis (CEBPA and BACH1) were significantly enriched for DEGs between SH3TC2 high-expression and SH3TC2 low-expression group, suggesting that these TFs may be potential targets of SH3TC2 in AML (Figure 10).

**Table 3:** Association between SH3TC2-DT expression^a^ and clinical characteristics (logistic regression).

**Clinical characteristics**	**Total**	**OR in SH3TC2-DT**	***P*-value**
		**expression**	
Age (continuous)	148	1.01 (0.99–1.03)	0.218
Sex (male vs. female)	148	1.18 (0.62–2.26)	0.620
WBC (continuous)	148	1.01 (1.00–1.02)	0.042^*^
Blast percentage (continuous)	148	1.00 (0.98–1.012)	0.892
Mutation counts (continuous)	148	1.01 (0.96–1.07)	0.684
Cyto-risk (intermediate	112	4.40 (1.82–11.63)	0.002^**^
vs. good)			
Molecular risk (intermediate	108	4.35 (1.79–11.53)	0.002^**^
vs. good)			
Therapy (auto-SCT or	148	0.57 (0.29–1.10)	0.097
chemo vs. allo-SCT)			
NPM1 (WT vs. MUT)	148	0.55 (0.25–1.18)	0.128
DNMT3A (WT vs. MUT)	148	0.46 (0.20–1.00)	0.054
FLT3 (WT vs. MUT)	148	0.28 (0.13–0.60)	0.001^**^

a *Categorical dependent variable, higher or less than the median expression level. OR, odds ratio; SCT, stem cell transplantation; WT, wildtype; MUT, mutant*.

* P < 0.05;

** *P < 0.01*.

**Table 4:** Association between SH3TC2 expression^a^ and clinical characteristics (logistic regression).

**Clinical characteristics**	**Total**	**OR in SH3TC2**	***P*-value**
		**expression**	
Age (continuous)	148	1.02 (1.00–1.05)	0.024^*^
Sex (male vs. female)	148	1.06 (0.55–2.02)	0.869
WBC (continuous)	148	1.01 (1.00–1.02)	0.011^*^
Blast percentage (continuous)	148	0.85 (0.44–1.62)	0.620
Mutation counts (continuous)	148	0.98 (0.92–1.04)	0.487
Cyto-risk (intermediate	112	3.56 (1.49–9.05)	0.005^**^
vs. good)			
Molecular risk (intermediate	108	3.71 (1.56–9.48)	0.004^**^
vs. good)			
Therapy (auto-SCT or	148	0.89 (0.46–1.72)	0.739
chemo vs. allo-SCT)			
NPM1 (WT vs. MUT)	148	0.55 (0.25–1.18)	0.128
DNMT3A (WT vs. MUT)	148	0.54 (0.24–1.17)	0.121
FLT3 (WT vs. MUT)	148	0.17 (0.07–0.38)	3.550*e*-05^***^

a *Categorical dependent variable, higher or less than the median expression level. OR, odds ratio; SCT, stem cell transplantation; WT, wildtype; MUT, mutant*.

* P < 0.05;

** P < 0.01;

*** *P < 0.001*.

To validate our findings, we analyzed the BeatAML (Vizome) dataset and found that both SH3TC2-DT and SH3TC2 were significantly highly expressed in FLT3-mutant AML (Figure S4). Furthermore, another validation cohort GSE37642-GPL570 also showed that high expression of SH3TC2 was associated with poor OS in AML (*P* = 2.04e−02) (Figure S5).

## Discussion

Although FLT3 mutation is commonly found in adult AML, its prognostic significance is still controversial (1). The mechanism of mutant FLT3 activation in leukemogenesis has not been definitely confirmed. Further elucidating the potential genes that are associated with FLT3 mutation and prognosis of AML is of great significance. Mining transcriptomic data from TCGA dataset could help to find prognostic factors that possibly participate in cancer development or evolution.

In the present study, using the TCGA-LAML dataset, we identified the DElncRNAs and DEmRNAs between FLT3-mutant and FLT3-wildtype AML. Functional enrichment analysis revealed that DEmRNAs were enriched in Wnt signaling pathway, PI3K–Akt signaling pathway, and Ras signaling pathway. Wnt signaling pathway was reported to cooperate with FLT3-ITD in leukemic signal transduction (25), PI3K-Akt and Ras were considered as downstream molecular pathways of FLT3 in leukemogenesis (26). Our result confirmed the function of FLT3 mutation in AML pathogenesis as previously reported. Afterwards, we constructed co-expression modules and related them to clinical traits through WGCNA. Using multivariate Cox regression analyses, we constructed prognostic risk models of lncRNA and mRNA to identify hub DEGs associated with AML prognosis. The presence of both SH3TC2-DT and SH3TC2 in respective prognostic risk models promotes us to further analyze this divergent lncRNA/mRNA pair in AML dataset.

Divergent transcription is a common phenomenon that mammalian promoters initiate transcription on both sides with opposite directions. However, the biological function and importance are still poorly understood. The findings that divergent transcription is found in most actively transcribed genes and divergent lncRNA/mRNA pairs exhibit coordinated changes in transcription suggests that it might regulate gene transcription (23, 24). Our study revealed that SH3TC2-DT and SH3TC2 were also coordinately highly expressed in FLT3-mutant AML samples, suggesting that divergent transcription might regulate SH3TC2 expression to play a role in AML pathogenesis.

SH3TC2 is displayed as one of the most prevalent genes and prognostic factors across multiple tumor types (27, 28). In neuroblastoma, lower SH3TC2 expression level was associated with MYCN amplification and poor survival (29). In diffuse large B cell lymphoma, SH3TC2 was identified as a signature gene of the poor prognostic CD5^+^ activated B-cell like (ABC) subtype (30). However, the prognostic value of SH3TC2 expression in AML has not been well-understood. In our study, we found that high expression of SH3TC2-DT and SH3TC2 were both associated with poor OS, FLT3 mutation, high WBC count, and intermediate cytogenetic and molecular–genetic risk in AML. These results suggest the association of the SH3TC2-DT/SH3TC2 gene pair with the proliferation function of FLT3 mutation. The association with high WBC count and intermediate genetic risk contributes to explanation of high SH3TC2-DT/SH3TC2 expression in relation to poor OS.

Although SH3TC2 expression is prevalent in multiple types of tumors, the function of SH3TC2 in cancer development or therapeutic resistance is less understood. The adjacent gene miR-584 is located in the first intron of the SH3TC2 gene and was reported to function as a tumor suppressor gene for glioma (31) and clear cell renal cell carcinoma (32), but to induce migration in breast cancer through TGF-β (33). We found that the phenotype of SH3TC2-DT/SH3TC2 high expression was enriched in HSC/LSC and CML quiescence gene sets by GSEA. TFs associated with stemness or leukemogenesis may be potential targets of SH3TC2. Our study suggests that the SH3TC2-DT/SH3TC2 gene pair may be associated with the stemness or quiescence of FLT3-mutant LSCs. Further study to elucidate the pathologic function of SH3TC2-DT/SH3TC2 in FLT3-mutant AML is probably valuable.

In the validating BeatAML dataset, the SH3TC2-DT/SH3TC2 gene pair was also highly expressed in FLT3-mutant AML. However, we did not find the correlation between high expression of this gene pair and OS. AML samples of this dataset are heterogeneous, including both *de novo* and secondary, both chemotherapy-treated and palliative therapy-treated, which might lead to the unexpected result. Thus, we turned to the GEO dataset GSE37642-GPL570 and found that high expression of SH3TC2 was associated with poor OS. Overall, SH3TC2-DT/SH3TC2 expression may be a possible biomarker to further optimize the prognosis of FLT3-mutant AML, but larger AML cohorts for further study are needed.

In summary, through integrated bioinformatic analyses, we identified hub lncRNAs and mRNAs associated with FLT3 mutation and AML prognosis. Among them, high expression of the SH3TC2-DT/SH3TC2 gene pair was found in FLT3-mutant AML and associated with poor prognosis, high WBC count, and intermediate genetic risk. The correlation of SH3TC2-DT/SH3TC2 expression with stemness, quiescence, and leukemogenesis suggests a possible role for this divergent gene pair in FLT3-mutant LSCs. Overall, our findings would help to better understand the possibly leukemogenic mechanisms of FLT3-mutant AML and to find possible candidate genes for prognostic and therapeutic usage.

## Data Availability Statement

The datasets generated for this study can be found in The Cancer Genome Atlas database (https://portal.gdc.cancer.gov/), Vizome (http://www.vizome.org/), and GEO (GSE37642-GPL570, https://www.ncbi.nlm.nih.gov/geo).

## Author Contributions

PY performed research, collected, analyzed, interpreted data, and wrote the manuscript. HL interpreted data and provided support. XS interpreted data, provided support, and edited the manuscript. ZP conceived the concept, designed the studies, performed research, collected, analyzed, interpreted data, wrote the paper, and took responsibility for the whole manuscript. All authors read and approved the final manuscript.

## Conflict of Interest

The authors declare that the research was conducted in the absence of any commercial or financial relationships that could be construed as a potential conflict of interest.
