# Supplementary material for: High Expression of the SH3TC2-DT/SH3TC2 Gene Pair Associated With FLT3 Mutation and Poor Survival in Acute Myeloid Leukemia: An Integrated TCGA Analysis
Source: Front Oncol. 2020 Jun 19;10:829. doi: 10.3389/fonc.2020.00829 (PMC7318790; doi:10.3389/fonc.2020.00829)
Supplement: Supplementary file 1 [file Data_Sheet_1.docx]

**High expression of SH3TC2-DT/SH3TC2 gene pair associated with FLT3 mutation and poor survival in acute myeloid leukemia: an integrated TCGA analysis**

Pengfei Yu,^1,2^ Haifeng Lan,^1^ Xianmin Song,^3^ Zengkai Pan^3,4^

^1^ Department of Hematology, Shanghai East Hospital, Tongji University School of Medicine, Shanghai, China.

^2^ Institute of Virology, Hannover Medical School, Hannover, Germany

^3^ Department of Hematology, Shanghai General Hospital Affiliated to Shanghai Jiao Tong University, Shanghai, China.

^4^ Department of Hematology, Hemostasis, Oncology, and Stem Cell Transplantation,

Hannover Medical School, Hannover, Germany

**Correspondence author:**

Zengkai Pan, PhD

Department of Hematology, Shanghai General Hospital Affiliated to Shanghai Jiao Tong University, Shanghai, China.

No. 85, Wujin Road, Shanghai 200080, P.R. China

Email: panzengkai@hotmail.com

Xianmin Song, MD

Department of Hematology, Shanghai General Hospital Affiliated to Shanghai Jiao Tong University, Shanghai, China.

No. 85, Wujin Road, Shanghai 200080, P.R. China

Email: shongxm@sjtu.edu.cn

Supplementary Tables and Figures


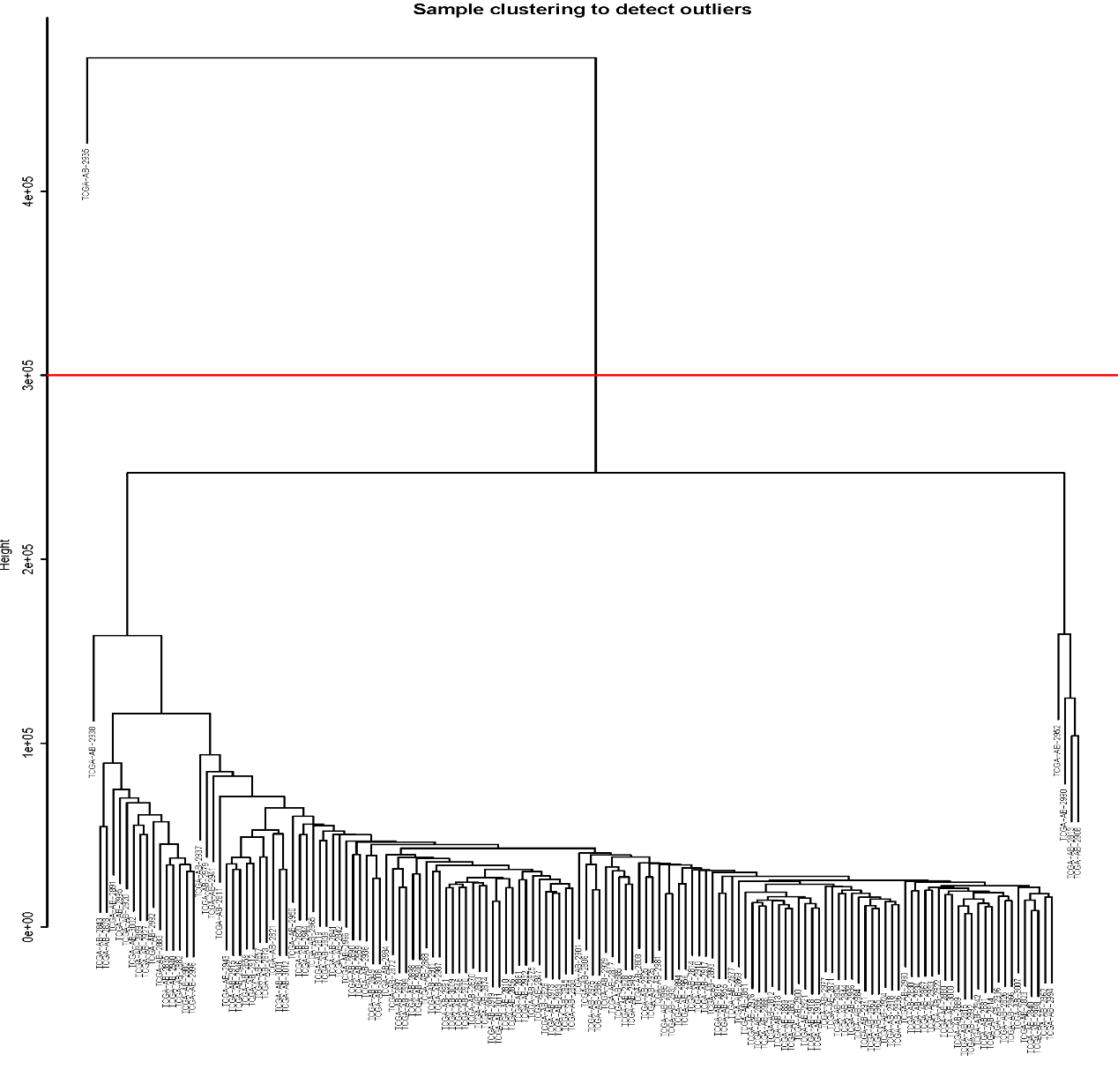


**Fig.S1. Sample clustering to detect outliers by “hclust” method.**

**
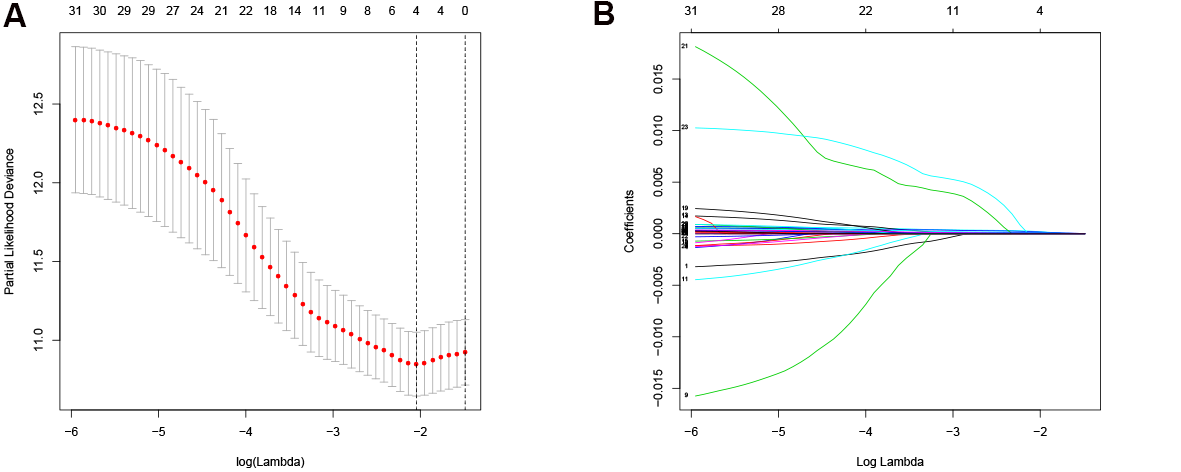
**

**Fig.S2. Feature selection by LASSO regression model**. (A) LASSO coefficient profiles of the 31 mRNAs from yellow module for overall survival. (B) Tuning parameter (lambda) selection in LASSO regression using 10-fold cross-validation via minimum criteria for overall survival.


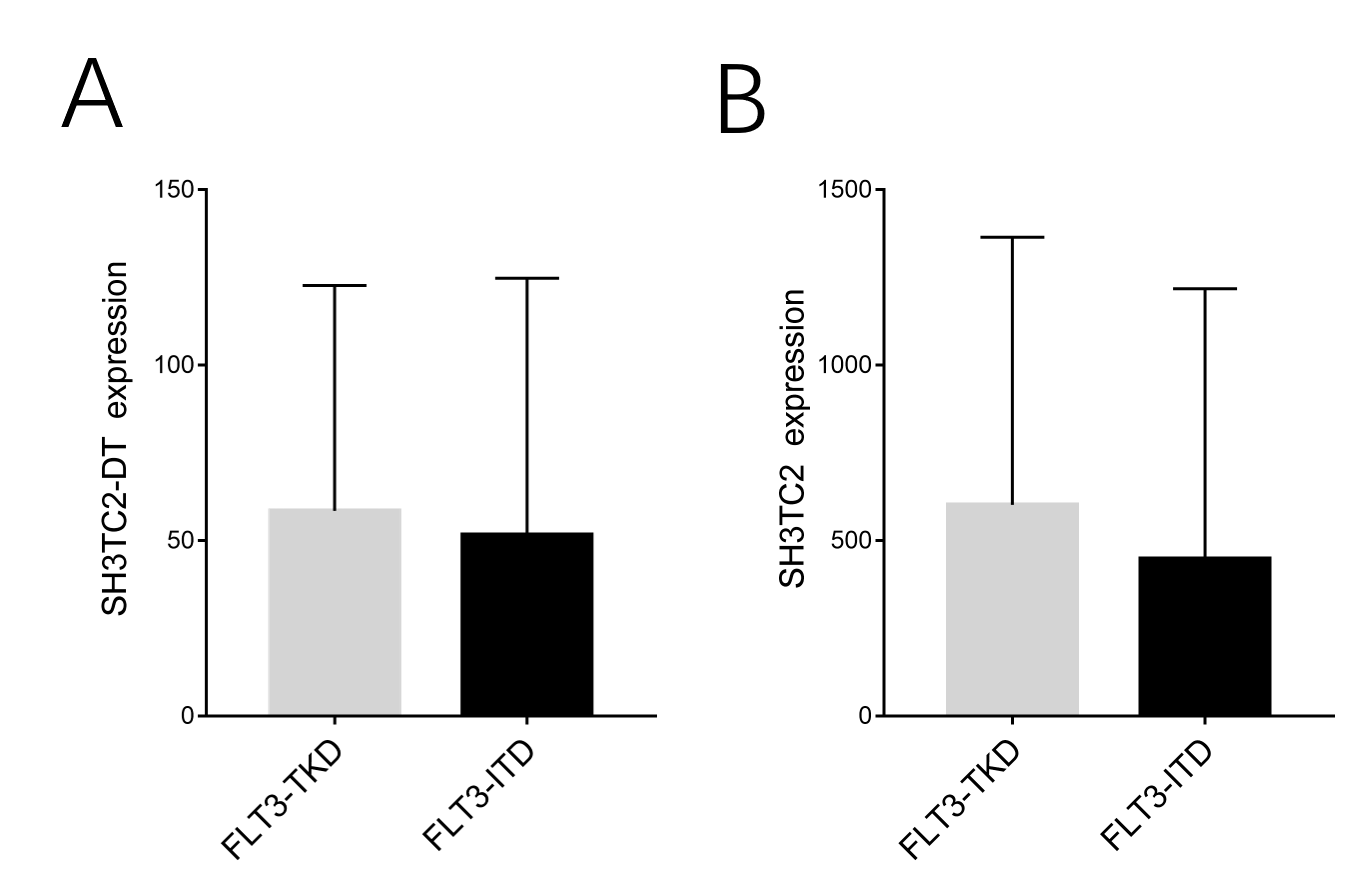
 **Fig.S3. Expression of SH3TC2-DT and SH3TC2 in FLT3-TKD and FLT3-ITD AML patients.** (A) The mRNA expression of SH3TC2-DT in FLT3-TKD and FLT3-ITD patients. (B) The mRNA expression of SH3TC2 in FLT3-TKD and FLT3-ITD patients. No statistical significance was found.

**
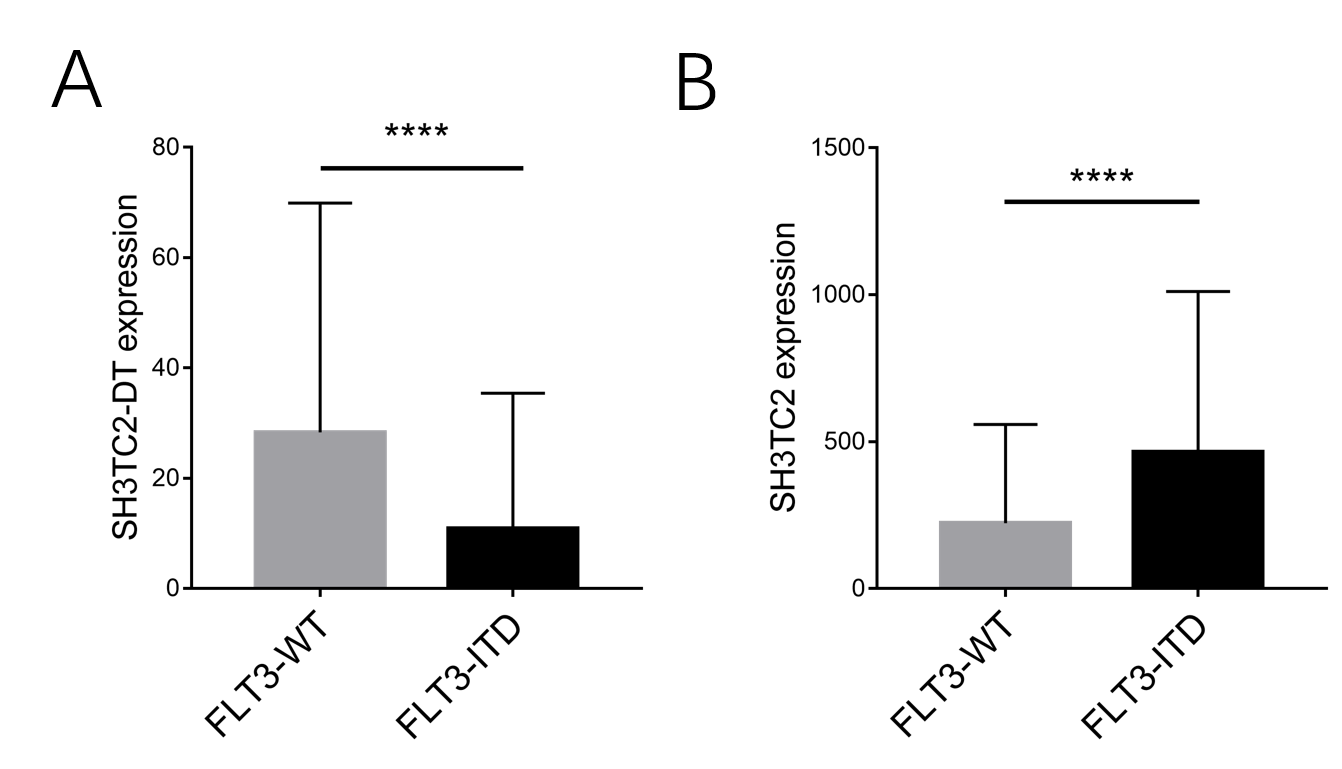
**

**Fig.S4. Expression of SH3TC2-DT and SH3TC2 in BeatAML dataset.** (A) The normalized RNA-sequencing expression value of SH3TC2-DT in FLT3-WT (N = 351) and FLT3-ITD (N = 109) AML samples. (B) The normalized RNA-sequencing expression value of SH3TC2 in FLT3-WT (N = 351) and FLT3-ITD (N = 109) AML patients.

**
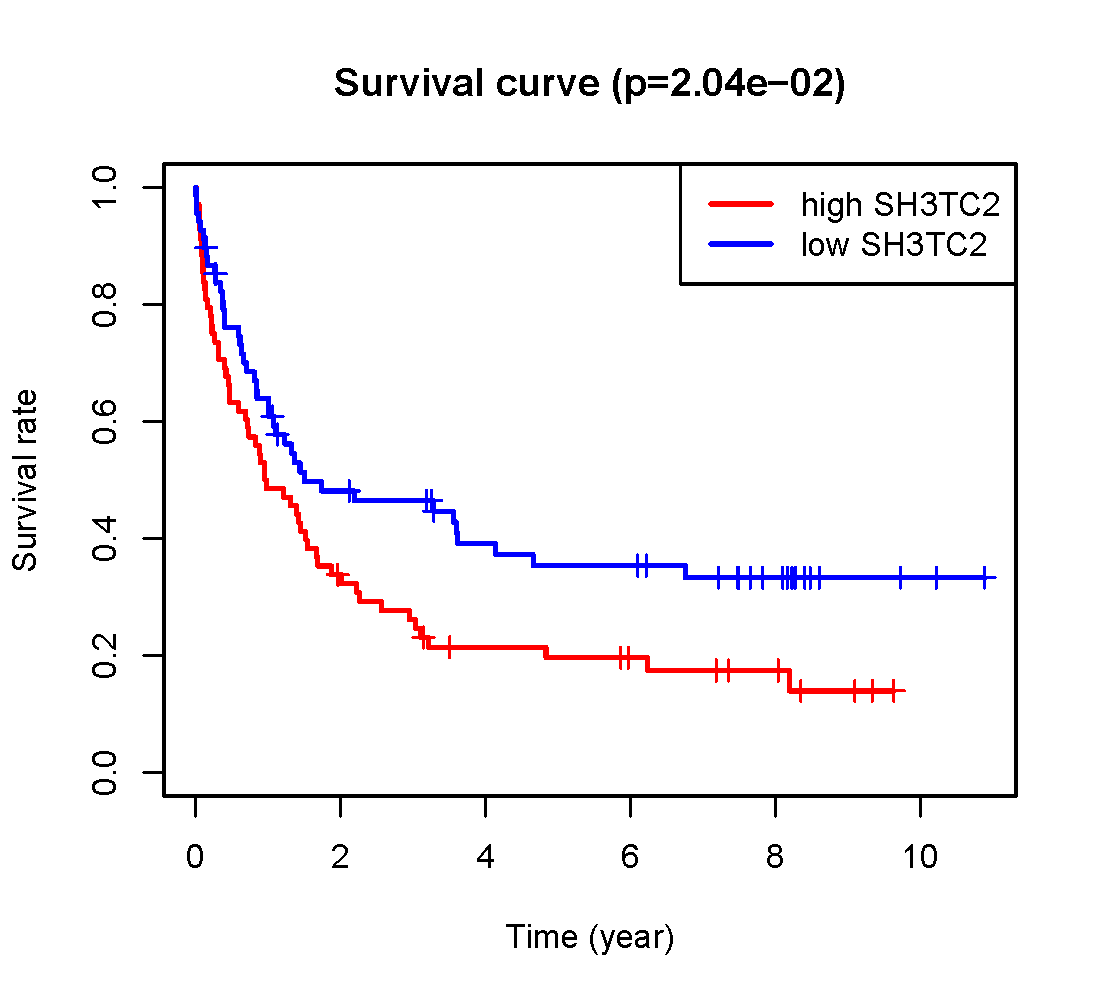
**

**Fig.S5. The association of SH3TC2 expression with overall survival in** **GSE37642-GPL570 validating dataset.** Kaplan-Meier curve to compare the overall survival between high- (N = 68) and low- (N = 68) SH3TC2 expression AML samples. The 136 AML samples were separated into two groups according to the median of SH3TC2 expression level.

**Table S1. A total of 12 prognostically significant lncRNAs from yellow module identiﬁed by univariate Cox regression analysis.**

HR, Hazard Ratio; 95% CI, 95% confidence interval.

| lncRNA | HR | 95% CI of HR | P value |
| --- | --- | --- | --- |
| SH3TC2-DT | 1.007252909 | 1.003609538-1.010909506 | 9.28E-05 |
| LINC00982 | 1.00065588 | 1.000300525-1.00101136 | 0.000296822 |
| LINC00899 | 1.000383755 | 1.000127839-1.000639738 | 0.0032904 |
| GRM7-AS1 | 1.107511819 | 1.03363969-1.186663439 | 0.00373896 |
| MIR155HG | 1.000670593 | 1.000196337-1.001145073 | 0.005577424 |
| AC005392.2 | 1.005275825 | 1.001359993-1.00920697 | 0.008230485 |
| LINC01132 | 1.008759738 | 1.000925776-1.016655015 | 0.028336579 |
| AL133353.1 | 1.018123074 | 1.001524784-1.034996448 | 0.032222451 |
| AF064858.1 | 1.000248394 | 1.000020861-1.000475979 | 0.032380173 |
| LINC01979 | 1.004910808 | 1.000334346-1.009508207 | 0.035421565 |
| AC103702.1 | 1.000650296 | 1.000032783-1.00126819 | 0.039012959 |
| DLGAP1-AS3 | 1.00304897 | 1.000059645-1.00604723 | 0.04559428 |

**Table S2. A total of 31 prognostically significant mRNAs from yellow module identiﬁed by univariate Cox regression analysis.**

| mRNA | HR | 95% CI of HR | P value |
| --- | --- | --- | --- |
| TMEM273 | 1.000322178 | 1.000174759-1.000469619 | 1.84E-05 |
| PRDM16 | 1.000375775 | 1.000192802-1.000558782 | 5.68E-05 |
| SH3TC2 | 1.00069841 | 1.00035196-1.001044981 | 7.76E-05 |
| LCT | 1.001845363 | 1.000924346-1.002767227 | 8.54E-05 |
| ENPP2 | 1.000602399 | 1.000279198-1.000925704 | 0.000258597 |
| CCDC113 | 1.005502524 | 1.002265563-1.00874994 | 0.0008514 |
| ATRNL1 | 1.010465035 | 1.004259859-1.016708551 | 0.000924646 |
| TRIM16 | 1.000986167 | 1.000400644-1.001572033 | 0.000960909 |
| LDLRAD2 | 1.000479654 | 1.000190471-1.00076892 | 0.001149171 |
| MPZL2 | 1.001537744 | 1.000607037-1.002469317 | 0.00119824 |
| HOXB6 | 1.0005057 | 1.000198081-1.000813415 | 0.001271528 |
| SCHIP1 | 1.000405159 | 1.000128145-1.000682249 | 0.004146185 |
| ARHGEF5 | 1.004973772 | 1.001559066-1.00840012 | 0.004275912 |
| CAMK2A | 1.022522506 | 1.006494251-1.038806009 | 0.005727404 |
| NLRP2 | 1.000362734 | 1.000103604-1.000621931 | 0.0060745 |
| CCL1 | 1.002230375 | 1.000580498-1.003882972 | 0.008041267 |
| H2AFY2 | 1.000774249 | 1.000200136-1.001348691 | 0.008206067 |
| GLI2 | 1.000465932 | 1.000115253-1.000816735 | 0.009207097 |
| APOL4 | 1.000397675 | 1.000086114-1.000709333 | 0.012356471 |
| HOXA7 | 1.001032707 | 1.000220638-1.001845435 | 0.012675511 |
| PBX3 | 1.000095298 | 1.000019315-1.000171287 | 0.013963089 |
| PDGFD | 1.000700761 | 1.000115093-1.001286772 | 0.019013592 |
| HOXB2 | 1.000270217 | 1.000037249-1.00050324 | 0.023003221 |
| HOXB5 | 1.000252534 | 1.000033024-1.000472092 | 0.024141984 |
| LCN8 | 1.00208006 | 1.000232393-1.00393114 | 0.027332382 |
| TRIM15 | 1.003441348 | 1.000264829-1.006627955 | 0.033699502 |
| GOLGA8B | 1.000130576 | 1.000005316-1.000255852 | 0.041038288 |
| CACNG4 | 1.000884206 | 1.00002602-1.001743129 | 0.043444089 |
| FAM47E-STBD1 | 1.04085063 | 1.000402706-1.082933929 | 0.047717732 |
| REN | 1.007318535 | 1.00007029-1.014619314 | 0.047811489 |
| FAM47E | 1.000927213 | 1.000002167-1.001853115 | 0.049465493 |

HR, Hazard Ratio; 95% CI, 95% confidence interval.
